# Old Antibiotics Can Learn New Ways: A Systematic Review of Florfenicol Use in Veterinary Medicine and Future Perspectives Using Nanotechnology

**DOI:** 10.3390/ani13101695

**Published:** 2023-05-19

**Authors:** Emilia Trif, Constantin Cerbu, Diana Olah, Sergiu Dan Zăblău, Marina Spînu, Adrian Valentin Potârniche, Emoke Pall, Florinel Brudașcă

**Affiliations:** Department of Infectious Diseases, Faculty of Veterinary Medicine, University of Agricultural Sciences and Veterinary Medicine Cluj-Napoca, Calea Mănăştur nr. 3-5, 400372 Cluj-Napoca, Romania

**Keywords:** florfenicol, alternative drug delivery, antibiotic-loaded nanoparticles, nanoscience

## Abstract

**Simple Summary:**

Florfenicol is a bacteriostatic antibiotic that is primarily used in veterinary medicine to treat a range of diseases in farm and aquatic animals. This synthetic analog of thiamphenicol and chloramphenicol works by inhibiting ribosomal activity, thereby disrupting bacterial protein synthesis, and has been proven in its effectiveness against a variety of Gram-positive and Gram-negative bacterial groups. Additionally, florfenicol has been found to possess anti-inflammatory properties and reduce immune cell proliferation and cytokine production. However, the inappropriate use of florfenicol has led to concerns about resistance genes, and its low solubility in water has made it difficult to formulate aqueous solutions using organic solvents. This review aims to synthesize the various applications of florfenicol in veterinary medicine, explore the potential use of nanotechnology to improve its effectiveness and analyze the advantages and limitations of such approaches. This review draws on data from scientific articles and systematic reviews found in multiple databases.

**Abstract:**

Florfenicol is a broad-spectrum bacteriostatic antibiotic used exclusively in veterinary medicine in order to treat the pathology of farm and aquatic animals. It is a synthetic fluorinated analog of thiamphenicol and chloramphenicol that functions by inhibiting ribosomal activity, which disrupts bacterial protein synthesis and has shown over time a strong activity against Gram-positive and negative bacterial groups. Florfenicol was also reported to have anti-inflammatory activity through a marked reduction in immune cell proliferation and cytokine production. The need for improvement came from (1) the inappropriate use (to an important extent) of this antimicrobial, which led to serious concerns about florfenicol-related resistance genes, and (2) the fact that this antibiotic has a low water solubility making it difficult to formulate an aqueous solution in organic solvents, and applicable for different routes of administration. This review aims to synthesize the various applications of florfenicol in veterinary medicine, explore the potential use of nanotechnology to improve its effectiveness and analyze the advantages and limitations of such approaches. The review is based on data from scientific articles and systematic reviews identified in several databases.

## 1. Introduction

**Figure 1:**
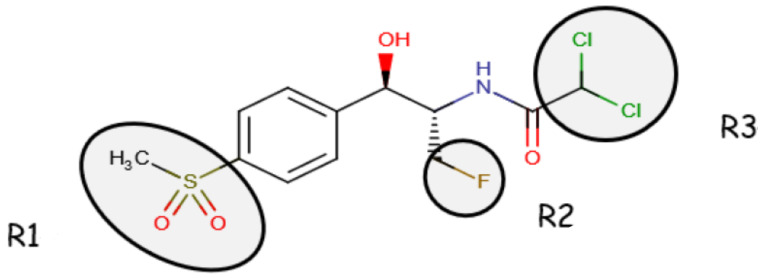
Chemical structure of FF (compared to Chloramphenicol, R2- hydroxyl group replaced by fluorine atom and R1-*p*-nitro group and the sulfomethyl group [2]) (Source for the modified figure: National Center for Biotechnology Information (2023). Pub Chem Compound Summary for CID 156406, Florfenicol amine).

Florfenicol (d-(threo)-1-(methylsulphoylphenyl)2-dichloroacetamide-3-floro-1-propanol) (FFC) is a synthetic antibiotic [1] included in the class of amphenicols (along with chloramphenicol, thiamphenicol, and azidamfenicol), with placement made by their phenylpropanoid structure [2]. FFC is the only antibiotic from the abovementioned class that was exclusively designed for veterinary therapeutics since it complements the shortcomings of chloramfenicol: an antimicrobial that lost its approval because of its toxic secondary effects on humans through food products of animal origin [3]. It is an antibiotic with a bacteriostatic action, provided by its binding to the bacterial 50S ribosomal subunits [2] and inhibiting the peptidyl transferase, which is an enzyme indispensable for protein synthesis [4]. FFC (chemical structure illustrated in Figure 1) is a derivative analog of chloramphenicol and thiamphenicol (chemical structure of florfenicol-related substances illustrated in Table 1 [5]) that presents only an active D-threo stereoisomer with an antimicrobial effect [5].

FFC is documented as effective against a large group of pathogenic bacteria, both aerobic and anaerobic, including Gram-positive and Gram-negative [6], and also against certain types of ryckettsia and chlamydia [7]. Its effects have lead to the development of medical applications for livestock, which are to be detailed further. Additionally, FFC has been demonstrated to possess an anti-inflammatory effect by inhibiting the NK-kB pathway in vitro [8]. Even though FFC complements the shortcomings of the antimicrobial substance from which it originates (one of the most important is that is not incriminated to the same extent as the occurrence of aplastic anemia [9]), it still presents certain limitations that could eventually led to the necessity of finding a replacement. Although florfenicol can be classified as a relatively new antibiotic (first reported use in 1990 in Japan) [7], its widespread use for both metaphylactic and prophylactic purposes [10] and as a growth promoter in aquaculture [11] has led to the premature development of resistance genes. This phenomenon affects both veterinary and human medicine, as bacteria with florfenicol-resistant genes have been identified among the human population, despite the fact that it is exclusively used in veterinary medicine [12]. This underscores once again the importance of rationally using antimicrobial substances in veterinary medicine [13], as this consequence affects all biomedical fields. The improper use of this antibiotic, such as underdosing, overdosing, or unjustified administration, may lead to an inability to obtain an appropriate therapeutic response in the future. Not only does the development of resistance over the time limit the usefulness of florfenicol in therapeutic applications of veterinary medicine, but its low solubility in water also presents a challenge [14]. This property makes it difficult to distribute this antibiotic in a stable and efficient form for oral administration, as it requires a prior process of solubilization in organic solvents [15]. In addition, florfenicol has been identified as having certain immunosuppressive potential through a significant reduction in immune cell proliferation: an effect that can have serious clinical and economic consequences [8]. For example, it can lead to a decreased humoral immune response in livestock (animals following vaccination), as well as a dose-dependent harmful effect on the reproductive system, especially in birds [16]. Despite all these considerations, florfenicol is still considered an effective antibiotic and is recommended for use in veterinary medicine. However, there is a need to improve its pharmacokinetics, pharmacodynamics, and routes of administration in order to increase its efficacy. Therefore, various biomedical techniques have been developed to address these issues for florfenicol as well as other antibiotics [17,18], through the application of nanotechnology, in order to obtain nanoscale applications for FFC. According to the Encyclopedia of Pharmaceutical Technology, nanostructures can be defined as solid colloidal particles that range in size from 1 to 1000 nm and serve as drug carriers, containing an active ingredient in a dissolved, entrapped, or encapsulated form, to which the active ingredient can be adsorbed or attached [19]. Novel forms of administering FFC, such as nanoemulsions [14] and polymeric nanoparticles made of natural polymers such as chitosan [20] or synthetic polymers such as PLGA [9], show great potential for enhancing the efficacy of this antibiotic due to their unique properties such as controlled release, stability, and targeting ability. For instance, polymeric nanoparticles based on PLGA are able to offer a sustained release of FFC and provide targeted delivery to specific cells or tissues, while nanoemulsions can improve the solubility and bioavailability of FFC. However, these novel delivery systems also present certain limitations and drawbacks, such as potential toxicity or challenges in achieving an optimal particle size and stability. These factors must be carefully considered and addressed in order to fully exploit the potential of nanotechnology for the improvement of the therapeutic use of FFC.

Florfenicol’s role in veterinary medicine is significant and warrants regular evaluation for its efficacy, safety, and further development potential. Despite the existing literature, the continuous influx of new research necessitates an updated, comprehensive review encompassing these latest findings. Centralizing all this information into a single review provides a clear snapshot of the current understanding of florfenicol use in veterinary medicine. It guides researchers and veterinarians to make informed decisions about its application across different animal species. Moreover, incorporating new research can illuminate the potential areas for future exploration and the development of this antibiotic, thereby reinforcing the importance of this review.

## 2. Materials and Methods

**Figure 2:**
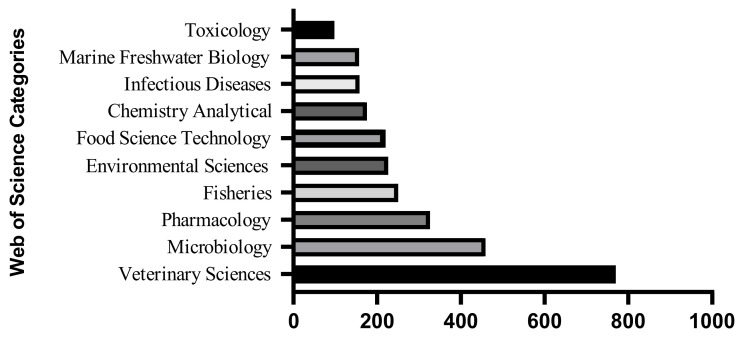
Results generated by the Web of Science database searching for the key word “Florfenicol”.

This review is based on scientific papers and systematic reviews identified on multiple databases (e.g., Web of Science, PubMed). Increased interest among researchers, mainly from veterinary sciences (as illustrated in Figure 2), was observed since the first paper published regarding the pharmacokinetics of florfenicol in veal calves in 1986 [21] until it reached its highest number in 2021 (228 publications that include three reviews of the literature).

**Figure 3:**
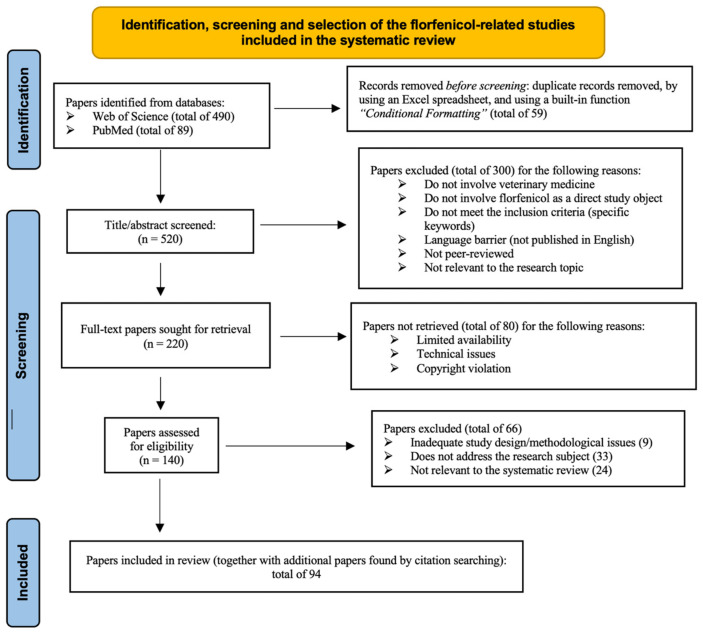
Prisma 2020 flow diagram including searches in Web of Science and PubMed databases.

The collected data (Figure 3) were further analyzed using GraphPad Prism 9.3.0 in order to illustrate the study selection process. The diagrams provide a visual representation of the number of articles identified, screened, assessed for eligibility, and included in the present study.

**Figure 4:**
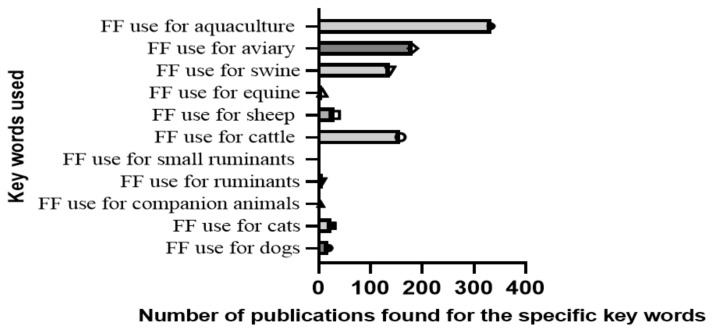
A graphical analysis of publication count by specifically used keywords.

A number of 39 review articles with over 200 research articles and short communications were categorized based on specific keywords to gather relevant data on the use of florfenicol in various animal species, as well as studies on novel techniques to enhance its delivery as an antimicrobial agent. Since it represents a high interest in veterinary therapeutics, keywords were oriented by the species for which florfenicol is mostly used, unexpectedly indicating widespread interest in the use of this antibiotic in aquaculture (Figure 4).

In order to help readers identify the practical uses of this antibiotic, its current use in different veterinary pathologies, and the latest improvement techniques, along with their advantages and limitations, the findings of this systematic review were consolidated and presented in a summarized table format.

## 3. Classification of Medical Uses of FFC Based on Targeted Species

### 3.1. FFC Use in Companion Animals

In dogs and cats, florfenicol is frequently used for the treatment of dermatological conditions of bacterial etiology, such as external otitis (a pathology not to be neglected since it represents the third most common diagnosis in companion animals [22]). Randomized clinical trials confirmed the efficacy of the topical application of florfenicol combined with terbinafine (through the available commercial form- topical gel) in this pathology [23,24]. Research articles, both from 2008 [25] and 2015 [1], conclude that florfenicol could represent a useful therapeutical option for other bacterial infections in dogs, but this clinical approach should be made carefully since there are no available studies regarding FF efficacy and its possible toxicity [1,26].

### 3.2. FFC Use in Rabbits

FFC is known to be effective against various digestive and respiratory tract infections [27], including blocking the growth of *Streptococcus agalactiae*, a pathogen that causes severe and antibiotic-resistant infectious diseases in domestic rabbits [28,29]. Additionally, since the simultaneous use of coccidiostats and antibiotics is a common practice in rabbit care, both for preventing and treating parasitic and infectious diseases, FFC can be associated with three different coccidiostats, but in this documented case, its elimination was reported as strongly accelerated [30].

### 3.3. FFC Use in Ruminants

FFC is mainly used for large ruminants in the treatment of bovine respiratory diseases caused by etiological agents such as *Pasteurella multocida*, *Mannheimia haemolytica*, or *Histophilus somni* [6], which are included in the undifferentiated fever syndrome of the bovine: a syndrome that is frequently associated with other pathologies such as BVD (bovine viral diarrhea) [31]. Even though there are other therapeutical alternatives for this syndrome, FFC was presented as the most preferable option for treatment when comparing the advantages versus costs [32]. Regarding its pharmacokinetics, previous data state that FFC presents good bioavailability when administered parenterally and orally (except when given as a milk replacer). This concludes that a treatment protocol that includes one or two administrations per day (depending on the MIC of the involved pathological agent) could be very effective [21].

In cattle, florfenicol products are also intended for therapeutic use in acute interdigital necrobacillosis, presenting good antimicrobial activity against *Fusobacterium necrophorum* and *B. melaninogenicus* [33], but also in the case of keratoconjunctivitis produced by *Moraxella bovis* [6]. Despite the fact that neither florfenicol nor chloramfenicol was approved for digestive tract infections produced by bacteria from the *Escherichia* genus, resistance genes were found in clinical isolates from cattle with diarrheal syndrome [34], highlighting the rapid emergence of bacterial resistance against the amphenicols antimicrobial group. To consolidate this hypothesis, as a result of a study performed on a commercial farm, it was concluded that even though the administration of florfenicol in dairy calves did not significantly affect the soil microbiome, by direct contact with fecal matter, grazing, and other farming activities, it led to an overall rise in the antibiotic resistance genes present at this level (resistome). Many of these genes have the potential to be transferred, increasing the risk of the soil-animal and later on animal–human transmissions in antibiotic-resistant genes [35].

Although FFC is a broad-spectrum antibiotic approved by the Food and Drug Administration (FDA) for use in cattle, swine, and fish and by the European Medicines Agency (EMA) for use in cattle, sheep, and fish, maximum residue limits have been extrapolated in all species that produce food for human consumption, including goats, due to the limited number of products available for therapeutic approaches in this species [36]. Although, by extrapolation, florfenicol is approved by the FDA and EMA for use both in large and small ruminants, neither agency has approved its use in lactating animals, even though there are no tolerance values allowed for milk, and its use in the mentioned situations was conducted outside of the manufacturer recommendations made (extra-label/off-label) [36,37,38].

Based on the clinical score and complete remission, as well as the laboratory results obtained from performing antibiograms, FFC can be a good choice when treating respiratory infections in small ruminants caused by *Mannheimia haemolytica* [39]. Additionally, a study was identified that tested the efficacy of florfenicol in its therapeutic approach to caseous lymphadenitis in sheep and goats caused by *Corynebacterium pseudotuberculosis*. A comparison of the control groups and those who received florfenicol therapy showed an improvement in clinical scores, suggesting the effective treatment and maintenance of remission in caseous abscesses [39]. Considering these findings, as well as the clinical and bacteriological results, FFC may be effective in combating this pathology, but more studies are needed in order to confirm this [39]. Given the intracellular localization of the bacteria responsible for caseous lymphadenitis and the formation of biofilm in natural infections [40], which reduces the effectiveness of drugs, the successful use of florfenicol in alleviating this pathology and the clinical recovery represents aspects that can counteract the costs involved, make this antimicrobial beneficial for disease management at the herd level [39].

### 3.4. FFC Use in Equine

Even though it complements the limitations of chloramfenicol (estimated half-life of 1 h and consecutively the need for multiple dose administration for 24 h in order to achieve an optimal plasma concentration) [41], FFC is not the first option when needing an antimicrobial agent in equine species. Adverse reactions such as modifications in stool consistency and modified smells (signs that can indicate incipient enterocolitis) [42] and also modified biochemical parameters (increased bilirubin levels in plasma) have been documented. Additional safety tests were considered necessary concerning the use of florfenicol in equidae, but since the study was published in 1996 [41], no additional data were identified.

### 3.5. FFC Use in Swine

In pigs, FFC is used for the treatment of bacterial respiratory diseases caused by agents such as *Actinobacillus pleuropneumoniae*, *Pasteurella multocida*, *Bordetella bronchiseptica*, and *Salmonella choleraesuis* [6,43]. Although it has high efficacy against these pathogens, FF should be used with caution in this species as it can cause changes in the intestinal microbiome, promoting the emergence of resistance genes (plasmid-mediated resistance or cross-resistance) [44]. Additionally, the synergistic effect of florfenicol and macrolide antimicrobials (such as tilmicosin) has been identified for the treatment of respiratory diseases in pigs [45]. A recent study presented florfenicol to be a reliable option for the treatment of arthritis in pigs caused by *Streptococcus suis*, but with dose adjustment dependent on the minimum inhibitory concentration for the specific pathogen determined beforehand (the study showed favorable results with an initial dose of 30 mg/kg body weight), followed by maintenance doses of 15 mg/kg [46].

### 3.6. FFC Use in Aviary Medicine

FFC is an antibacterial medication that is used quite frequently in bird pathology, but due to its low solubility in water, administering it in drinking water can lead to a considerable variation in the concentration of the active substance among treated individuals [15]. However, administering an individual injectable preparation can have a negative effect on carcass quality. In addition, the porto-renal system in birds can reduce the bioavailability of this antimicrobial when administered in the caudofemoral region of the body [15]. Moreover, it has been used over time for prophylactic purposes in order to prevent the gastrointestinal and respiratory infections caused by microorganisms that are sensitive to florfenicol, such as *Ornithobacterium rhinotracheale*, *Mannheimia haemolytica*, *Salmonella typhi*, *Pasteurella multocida*, *Enterobacter cloacae*, *Escherichia coli*, *Haemophilus somnus*, *Klebsiella pneumoniae*, *Shigella dysenteriae*, and *Staphylococcus aureus* [47,48,49,50]. Following the existence of a suspicion, pharmacovigilance studies were carried out consisting of a five-day treatment at a dose of 10 mg/kg (both males and females treated metaphylactically) to manage *Escherichia coli* infection [16]. The test revealed a severe decrease in egg hatching, resulting in embryonic death, as a result of using the antimicrobial substance outside the manufactures’ recommendations. It was thus recommended to limit its use in breeding flocks, except in special situations where the value of the breeding flock indicated that their clinical condition was more important than a decrease in egg fertility.

### 3.7. FFC Use in Aquaculture

Data from previous pharmacokinetic and in vitro sensitivity studies have shown florfenicol to be a good alternative to the most commonly used antimicrobial agents when treating bacterial infections in fish [51,52,53] since its first approval for use in aquaculture in the United States in 2000 [54]. Florfenicol can be used specifically to combat bacterial pathologies in rainbow trout caused by *Flavobacterium psychrophilum* [55]. Florfenicol has proven effective in treating furunculosis: a severe disease in salmonids caused by *Aeromonas salmonicida* [56]. Additionally, studies have demonstrated its superiority over thiamphenicol, chloramphenicol, and oxytetracycline in experimental infections with *Pasteurella piscicida* in carp, *Edwardsiella tarda* in Japanese perch, and *Vibrio anguillarum* in certain species of carp [53].

The effectiveness of florfenicol when treating sea bass pathologies caused by bacterial agents such as *Flavobacterium columnare* has subsequently been determined, significantly reducing mortality without producing the macroscopic lesions associated with florfenicol treatment [52].

### 3.8. Available Formulations for Clinical Use

The following table (Table 2) provides an overview of florfenicol use in veterinary medicine, classified by targeted species and the availability of commercial products, including their indications. It is important to note that the availability of commercial products may vary over time and across regions. Therefore, the information presented in this table reflects the availability of florfenicol products at the time of writing. This table aims to provide veterinarians and other animal health professionals with a quick reference guide on the use of florfenicol in veterinary medicine.

## 4. Limitations and Future Perspectives on Enhancing Florfenicol: Nanotechnology for Improved Clinical Delivery

Despite being widely used in veterinary medicine mainly due to its effectiveness against multiple bacterial infections in animals [6], there are several limitations that must be taken into consideration before using FFC as a primary treatment option. One of the major concerns is the potential development of antibiotic-resistant bacterial strains [58]. While the possibility of resistant genes development is notably lower compared to other antibiotics of the same amphenicol class [58,59], studies have indicated the fact that the administration of FFC induces the transmission and evolution of genes that confer resistance to multiple drug classes, providing a resistome group for environmental microorganisms [35]. The microbiome disturbance resulting from florfenicol treatment may induce microbiome-mediated colonization resistance and an increase in the risk of infection, which could lead to drastically reduced fecal microbiome diversity and recurrent drug-resistant infections [60,61]. In addition to the significant risk of developing antibiotic resistance, the use of FFC is also limited in specific animal species due to certain constraints. Table 3 presents an extensive overview of the limitations linked to the utilization of FFC across different animal species, as well as its potential improvements. Recognizing these limitations is critical for veterinarians and animal producers to determine the suitable applications of this antibiotic and to ensure the safety of animal products intended for human consumption.

To summarize, the above table brings attention to several drawbacks that are associated with the clinical use of florfenicol (bone marrow suppression, gastrointestinal side effects, residue accumulation, low solubility, and the emergence of antibiotic-resistant bacterial strains). Nevertheless, these harmful effects can be minimized by implementing certain strategies. The proposed approaches suggest reducing the dosage, altering the administration route, targeting specific sites, developing sustained-release drug formulations, and improving water solubility. Nanotechnology-based drug delivery systems (as outlined in Table 3) can facilitate the implementation of these solutions.

Nanobiotechnology, or nanotechnology in medicine, is a hybrid science that has emerged through the collaboration of two main advanced technologies: biotechnology and nanotechnology [77]. This has resulted in an increased ability to investigate levels previously unattained by carefully combining the effectiveness of biological materials with the rules of basic science to fabricate synthetic structures of tiny dimensions [67,78]. These applications have been integrated, as expected, into biomedical devices, as most biological systems are at the nanometer scale, alongside the devices and nanomaterials used exhibit increased biocompatibility [14,77]. Therefore, metallic, ceramic, polymeric, and composite nanomaterials have been extensively investigated for various biomedical applications, such as tissue engineering, targeted drug delivery systems, and biosensors [79]. Therefore, nanotechnology plays a central role in improving recent technologies in the field of pathology diagnosis as well as in designing and administering drugs, known as nanomedicine [80]. Nanomedicine can be defined as the understanding and restructuring of biomaterials down to the nanometer scale and can play a remarkable role in the controlled delivery and administration of active substances, with the aim of using them at predetermined rates and targeting a desired group of cells [81].

Nanoparticle drug development can potentially achieve therapeutic effects with minimal doses [82] and can be utilized in antimicrobial therapy thanks to their unique physicochemical properties [83]. These systems can address bioavailability needs and enable better-controlled release at targeted sites [84]. Consequently, nanostructures containing florfenicol have been created and assessed for their effectiveness as drug carriers in these innovative delivery systems. To provide an overview of the nanostructures and their characteristics, a table (Table 4) summarizing the synthesis methods together with their advantages and disadvantages has been compiled. This table includes various types of nanostructures, such as nanoemulsions, polymeric nanoparticles based on PLGA or other types of polymers, as well as chitosan or albumin. This table aims to provide a brief and comprehensive overview of a diverse range of nanostructures containing florfenicol, which was developed using nanobiotechnology.

## 5. Conclusions

In summary, florfenicol is a broad-spectrum antibiotic that is widely used in veterinary medicine, with proven effectiveness and safety. Yet, there is an ongoing need to optimize this drug to enhance its therapeutic benefits and minimize side effects. The surge in related publications underscores a growing interest in its potential and limitations and efforts to improve its efficacy. Nanotechnology has been identified as a promising method for augmenting florfenicol’s effectiveness, stability, and delivery. By altering the drug’s physical and chemical properties, it may be possible to address limitations related to solubility and bioavailability and even enhance activity against resistant bacteria. Nanocarriers could improve drug delivery and reduce toxicity, allowing for better treatment outcomes. However, nanotechnology has its own challenges, including potential toxicity, large-scale production difficulties, and higher manufacturing costs. Despite these obstacles, researchers persist in exploring new techniques and solutions to these challenges. Ultimately, nanotechnology’s application in enhancing florfenicol’s properties offers great potential for future veterinary medicine research, leading to improved animal health and reducing antimicrobial resistance risks.

## Figures and Tables

**Table 1:** Structure of florfenicol-related substances.

Amphenicol	R1	R2	R3
Florfenicol	-SO_2_CH_3_	-F	=Cl_2_
Chloramphenicol	-NO_2_	-OH	=Cl_2_
Thiamphenicol	-SO_2_CH_3_	-OH	=Cl_2_
Azidamfenicol	-NO_2_	-OH	H-R-N=N≡N

**Table 2:** Florfenicol use in veterinary medicine, classified by targeted species and the availability (at the time of the writing) of commercial products together with their indications.

Targeted Species	Targeted Pathology	Dose	Route of Administration	Possible Side Effects	Restrictions of Human Use of the Animal Products	Availability of a Commercial Product	Other Specifications	Source
Canine	Dermatological conditions- pyoderma, external otitis (associated with *Staphylococcus pseudintermedius* and *Malassezia pachydermatitis*)	10 mg/ administration 1–2 times a day (spray)	Oral administration Optic administration (local gel formulation) Intramusculary or subcutaneous Spray-on	Vomiting Increased liver enzymes Loss of hearing Weight loss	Not applicable	Osurnia^®^ Florfenicol 30% Alpharabi^®^ Simplera^®^ Floxy-Spray Max^®^	Associated with other active substances such as terbinafine and bethametasone acetate or gentian violet	[1,22,23,24,25]
Feline	Dermatitis of bacteriological etiology (bacterial groups susceptible to florfenicol such as *Staphylococcus pseudintermedius*, *Pseudomonas* spp.) External otitis	10 mg/ administration 1–2 times a day (spray)	Intramusculary or subcutaneous Spray-on	Vomiting Reduction in hearing Weight loss	Not applicable	Florfenicol 30% Alpharabi^®^ Floxy-Spray Max^®^	Associated with other anti-inflammatory substances or with gentian violet	[6,22]
Bovine	Bovine respiratory disease (BRD)- produced by *Mannheimia haemolytica*, *Pasteurella multocida* and *Histophilus somni*); Bovine interdigital phlegmon (produced by *Fusobacterium necrophorum* and *Bacteroides melaninogenicus*) Keratoconjunctivitis (produced by florfenicol-susceptible bacteria)	20 mg/kg body weight	Intramusculary (subcutaneous also possible)	Transient inappetence, diarrhea, local reaction, anaphylaxis, and collapse	Not suitable for use in lactating dairy calves (older than 20 months) or calves for veal production	Nurflor^®^ Nurflor Gold^®^ Resflor Gold^®^ Zeleris^®^ Florkem^®^ FlorfenCare^®^ Nifenicol^®^ Florfenicol 30% Alpharabi^®^ Selectan^®^ Fenflor^®^ Loncor^®^ Florfluject^®^ Taikocin^®^ 30%	Associated with Flunixin meglumine	[6,31,32,33,34,35,37]
Small ruminants	Respiratory infections caused by *Mannheimia haemolytica*. Caseous lymphadenitis produced by *Corynebacterium pseudotuberculosis*		Intramusculary	Transient inappetence, diarrhea, local reaction, anaphylaxis, and collapse	Not suitable for use in lactating animals	FlorfenCare^®^ Florfenicol 30% Alpharabi^®^	-	[36,37,39]
Aquatic animals- fish, carp, salmon, catfish	Carp red spot disease (Aeromonas infections, hemorrhagic septicemia), Furunculosis (salmon aeromonosis), vibriosis, Salmon *Haemophilus parasuis*, pseudomonosis, pasteurellosis, secondary bacterial infections after stress or after primary parasitic infections, enteric septicemia produced by *Edwardsiella ictaluri*	10 mg per 1 kg of fish body weight	Oral Orally, in water Oral-mixed with the feed	Not specified	Fish consumption is permitted after 8–16 days (depending on the water temperature)	Flovet 8%^®^ Florfenicol^®^ (Prilabsa) Aquaflor^®^ Florfen^®^ 15%	Not for use in fish under water temperature lower that 5 °C	[51,52,53,54,55]
Camelids	Respiratory diseases Interdigital necrobacillosis (foot rot) Pododermatitis	20 mg/kg body weight	Intramusculary or subcutaneous	Not reported	Not applicable	Nurflor^®^ Nurflor Gold^®^ Florfenicol 30% Alpharabi^®^	Off label use Prolonged half-life (31–100 h) after subcutaneous administration due to the peculiarity of the species	[57]
Pigs	Acute outbreaks of swine respiratory disease (produced by *Actinobacillus pleuropneumoniae*, *Pasteurella multocida*, *Bordetella bronchiseptica*), Glasser disease (*Haemophillus parasuis*) To increase weight gain and improve feed conversion	10–15 mg/kg body weight	Intramusculary Orally, in drinking water Oral-mixed with the feed	Vomiting Anorexia Decreased water consumption Redness in the perianal region	Not permitted for human consumption 18 days after administration	Nurflor^®^ Florkem^®^ FlorfenCare^®^ Florfenicol FP 10%^®^ Nifenicol ^®^ Selectan^®^ Norfenicol^®^ Florcrid 4% Premix^®^ Agraflor-200^®^ Flovet 8%^®^ Tiloflor^®^ premix Fenflor^®^ Introflor^®^ oral Florum^®^ 20% oral Taikocin^®^ 30% Taikosol^®^ 30%	Not recommended in swine intended for breeding Prohibited as growth promotor.	[6,43,45,46]
Aviary	Infectious serositis, Mycoplasmosis, Colibacillosis, Pasteurellosis, Fowl cholera, Infectious coryza, *Staphylococcus* infections, infectious produced by *Ornitobacterium rhinotracheale*), Gastrointestinal and respiratory tract infections (produced by *Actinobacillus* spp., *Pasteurella* spp., *Salmonella* spp., *Streptococcus* spp., *E. coli*) To increase weight gain and improve feed conversion	20 mg/kg body weight	Intramusculary Orally, in drinking water Oral-mixed with the feed	Increased water consumption Transient softening of the feces, diarrhea	Not permitted in poultry producing eggs for human consumption (withdrawal period for meat- 2 days)	FlorfenCare^®^ Florfenicol FP 10%^®^ Travipharma^®^ Florfenicol 10%^®^ Agraflor-200^®^ Flovet 8%^®^ Vimflor^®^ Tiloflor^®^ premix Introflor^®^ oral Coliflor^®^ VetSolution Florum^®^ 20% oral Taikosol^®^ 10%	Prohibited as growth promotor	[15,16,47,48,50]
Equine	Respiratory diseases, skin, and soft tissue infections (produced by florfenicol-susceptible bacteria)	22 mg/kg body weight	Intravenous Intramusculary Orally	Elevated bilirubin concentrations, diarrhea, possible enterocolitis	Not permitted for human consumption (withdrawal period for meat- 28 days)	Nuflor^®^ Resflor Gold^®^	Commercial products not labeled for equine use	[41,42]

**Table 3:** Potential improvements for overcoming species-specific limitations of FFC in clinical applications.

Targeted Species	Limitations in Clinical Use	Potential Improvement Using Nanotechnology	Source
Companion animals (dogs, cats, equine)	Risk of bone marrow suppression (leading to fatal plastic anemia, leukopenia, and thrombocytopenia); Gastrointestinal side effects (vomiting, diarrhea, colic); Unavailable for pregnant or lactating animals; More frequent anaphylactic reactions compared to other species. Loss of hearing, in case of topical use. Risk of developing antibiotic-resistant bacterial strains.	Significant dose reduction to mitigate the risk of bone marrow suppression (as this adverse effect may be dose-dependent); Modifying the dosage or frequency of administrations to minimize the occurrence of gastrointestinal side effects, anaphylactic reactions, and other side effects; Conducting safety tests to determine if lower doses can reduce the associated risks in pregnant or lactating animals; Exploring alternative routes of administration or utilizing controlled release formulations to overcome the adverse effects and enhance the safety profile;	[1,43,44,49,57,62,63,64,65,66,67]
Livestock (bovine, small ruminants, swine, aviary)	The possibility of residue accumulation in animal products-milk, meat, and eggs (which can exceed the Maximum Residue Limits-MRLs) and present a risk to human health; Gastrointestinal side effects (diarrhea, weight loss due to reduced feed intake); Administration route and dosage (subcutaneous injection of florfenicol can cause reactions at the injection site and tissue damage; oral administration may not result in effective therapeutic concentrations in the animal’s bloodstream); Slow clearance rate that may lead to potential toxicity; Poor water solubility can limit its effectiveness when administered via drinking water; Decreased egg production in laying hens and modified immune response, both dose-dependent; Impact on gut microbiota and metabolite composition on neonatal chickens, thus reducing their resistance to Salmonella infections; Risk of developing antibiotic-resistant bacterial strains.	Changing the route of administration or a controlled release may be able to overcome some of the adverse effects; Reducing or adjusting the dosage or frequency of administrations which can reduce the adverse effects listed; Using alternative routes of administration; Sustained-release drug formulations could allow for a slower and more controlled release of FFC over an extended period and could also be beneficial from a welfare point of view; Increased bioavailability and pharmacokinetics; Different approaches to improve the solubility of FFC in water, such as the use of solubilizing agents, solvents, or particle size reduction, in order to facilitate administration via drinking water.	[6,10,14,26,34,35,36,39,41,42,46,47,49,52,53,56,68,69,70,71,72,73,74,75,76]
Aquatic animals (fish, carp, salmon, catfish)	Presence of residues, which can have a negative impact on public health and the environment; Limited pharmacokinetic data; Withdrawal periods need to be respected before animals can be harvested for consumption; Higher risk of developing antibiotic-resistant bacterial strains since the water will contain unmetabolized active substances; Poor water solubility can limit its effectiveness and can only be administered into the feed pellets;	Reducing the dosage up to the limit at which the drug can manifest its therapeutic potential; Different approaches to improve the solubility of FFC in water, such as the use of solubilizing agents, solvents, or particle size reduction;	[51,54,55,56,74,75]

**Table 4:** Nanostructures loaded with florfenicol found in research of the literature at the time of writing, together with their synthesis method, size obtained and advantages and limitations.

Type of Nanostructure Loaded with Florfenicol	The Synthesis Method	Size	Identified Advantages	Identified Disadvantages	Source
Nano-emulsions	Catastrophic phase inversion	44.82 ± 1.04 nm	Uniform droplet size with narrow size distribution, improved bioavailability, faster absorption, and immediate dispersion of the drug; higher relative bioavailability compared to the control group; plasma drug concentrations maintained above the MIC against most common bacteria for up to 12 h in both nano-emulsions and control group.	Very low distribution of nanoparticles due to their large size, which can limit their diffusion through the capillary wall into the body tissues; a low volume of distribution for the nano-emulsion group than the control group, indicating that the drug was less widely distributed in the body tissues.	[14]
PLGA microparticles and nanoparticles	Emulsion-evaporation technique	115.32 and 130.83 nm	A narrow size distribution, which could enhance the effectiveness of the drug delivery system by ensuring consistent particle size and drug release rate; Complete drug release within 5 h, which can be useful in the treatment of acute infections; No interactions between the polymer and florfenicol, indicating that the drug was effectively entrapped in the particle matrix without any chemical modifications or degradation.	Low entrapment efficiency (20–25%), which may not be sufficient to achieve the therapeutic levels of the drug; Slower release rate (the microparticle system showed a slower diffusion rate after an initial rapid release, which could be disadvantageous for drugs that require an immediate release for short-term treatment); Unpredictable drug release and decreased effectiveness of the drug delivery system; Autocatalytic degradation	[9]
Bovine serum albumin-based nanoparticles (BSA nanoparticles)	Desolvation method	140 ± 39.5 nm	A high-stability colloidal system with suitable characteristics for drug delivery; Simple synthesis method and low costs; High drug loading of florfenicol in the nanostructures shows a promising nanocarrier for the administration of florfenicol for veterinary purposes; Sustained release, which is highly desirable to avoid multiple doses; Constant physicochemical properties effectively present potential as carriers for incorporation into injectable formulation;	Highly necessary in vivo tests to determine the safety, doses, pharmacological activity, and fate of this nano-system.	[85]
Nano-micelles based on chitosan-stearic acid (CS-SA)	Modified ultrasonication method	Not specified	Improved thermal stability of the nano-micelles, making its structural properties stable and not easy to decompose within 25–200 °C; Favorable shape, good loading capacity, and entrapment efficiency, sustained release characteristics in a simulated gastric juice (pH 1.2) and a simulated intestinal fluid (pH 6.80), which was in accordance with the main absorption sites of FF reported in the literature;	In vivo biological analysis is highly necessary to reach the correct conclusion about safety, doses, pharmacological activity, and the fate of the nano-system; Limited potential applications of nano-micelles.	[86]
Silica-based nanoparticles	Gelation method for silica nanostructures and loading the florfenicol into the aqueous solution obtained	179 nm	Increased solubility from 1 mg/mL to 30 mg/mL at around 95 °C, which could improve the drug’s bioavailability; Time-dependent release over a longer time compared to pure florfenicol, which may provide a controlled release and reduce the frequency of dosing; Larger particles consistg of tens of florfenicol-loaded silica nanoparticles, which may improve the stability of the formulation.	The delayed release of florfenicol from the silica nanoparticles (problematic where a quick onset of action is required); A burst release of nearly 80% within the initial 35 h, which may result in a higher concentration of florfenicol; Heating of the solution may be required to increase the solubility of florfenicol, which may not be feasible for some drug formulations or applications.	[87]
Nanoparticles based on composite nanogel	Ionic gelation method	200 nm	Enhanced concentration and residence time of drugs at the infection site, improving their antibacterial effect; Nanoscale size and uniform dispersion allow for easy passage through bacterial cell membranes and targeted release; The positive charge of nanogels can enhance the bactericidal effect through electrostatic interactions with bacterial membranes; Sustained release of drugs allows for targeted and long-lasting treatment; Stronger antibacterial activity compared to commercial florfenicol solution; Biosafety revealed no obvious side effects or toxic implications; Ideal therapeutic effect against cow mastitis;	Gelatinous consistency, which may not be suitable for all applications; No mention of comparison to other treatment options or cost-effectiveness compared to other treatments.	[88]

## Data Availability

All the relevant data is available in the manuscript.
